# Laboratory Inventory Management Engine (LIME): A free tool for managing laboratory inventories via barcode scanning and automatic cloud-based spreadsheet integration

**DOI:** 10.1371/journal.pone.0336412

**Published:** 2026-05-22

**Authors:** Tiffany M. Salinas, Leo Tornes, Christopher Solís

**Affiliations:** Department of Health, Nutrition, and Food Sciences, Florida State University, Tallahassee, Florida, United States of America; MIT, UNITED STATES OF AMERICA

## Abstract

Efficient inventory management remains a persistent challenge in research settings, where productivity and budget control are tightly linked to accurate tracking of consumables, reagents, and equipment for decision-making and successful research project execution. Many laboratories still rely on traditional methods, including handwritten logs or static spreadsheets, which are prone to human error and can result in stockouts, over-ordering, and inaccurate forecasting. Moreover, existing inventory systems often fall short in balancing affordability, convenience, and functionality. Here, the Laboratory Inventory Management Engine (LIME) is introduced as a system designed to bridge these deficiencies by leveraging accessible tools like in-the-cloud spreadsheet files and mobile devices. LIME uses a smartphone app to enable real time updates to the inventory log by scanning consumables QR codes and barcodes. The system allows inputting new items to be classified in predetermined catalog lists (e.g., 4º fridge, chemicals, antibodies, etc.). This dual smartphone and spreadsheet inventory prototype is anticipated to improve the operational efficiency of small academic research laboratories and early-stage startups to benefit their bottom lines.

## Introduction

Maintaining a laboratory inventory is an important but often underestimated task in research—yet it plays a critical role in the efficiency of labor. Inaccuracies in inventory tracking can lead to mistrials, delays, financial loss, and compromised data integrity [1]. Developing a reliable and efficient inventory system is essential for ensuring smooth experimental operations and maintaining optimal research quality.

Many laboratories track inventory in one of three ways, either through Excel® spreadsheets and Google Sheets®, sheets of paper, or via paid subscriptions to laboratory information management systems (LIMS) [2]. Systems brought forward through the use of LIMS, have paved way for advanced functionalities for tracking samples, managing data, and streamlining workflows. However, despite their growing popularity, these laboratory management systems may have shortcomings such as high cost, steep learning curves, and lack of customization [3]. These gaps have driven the continuous development of alternative solutions such as those that tap into the ever-changing advantageous environment brought by technology and mobile devices. Handheld mobile devices have the ability to act as scanners through the use of their camera features, opening the door for an inventory management system which parallels that seen in retail environments [4,5].

To address these gaps, this work introduces LIME: a cost-effective, mobile-centric, and spreadsheet-integrated system for inventory management. LIME enables users to submit inventory data through mobile forms with information seamlessly transmitted to Excel^®^ or Google Sheets^®^. This eases adoption as these tools already widely used and understood by the public and academic labs [2]. This dual-interface design harnesses the speed and convenience of mobile devices while retaining the flexibility and widespread use of spreadsheets.

## Structural features

**Fig 1:**
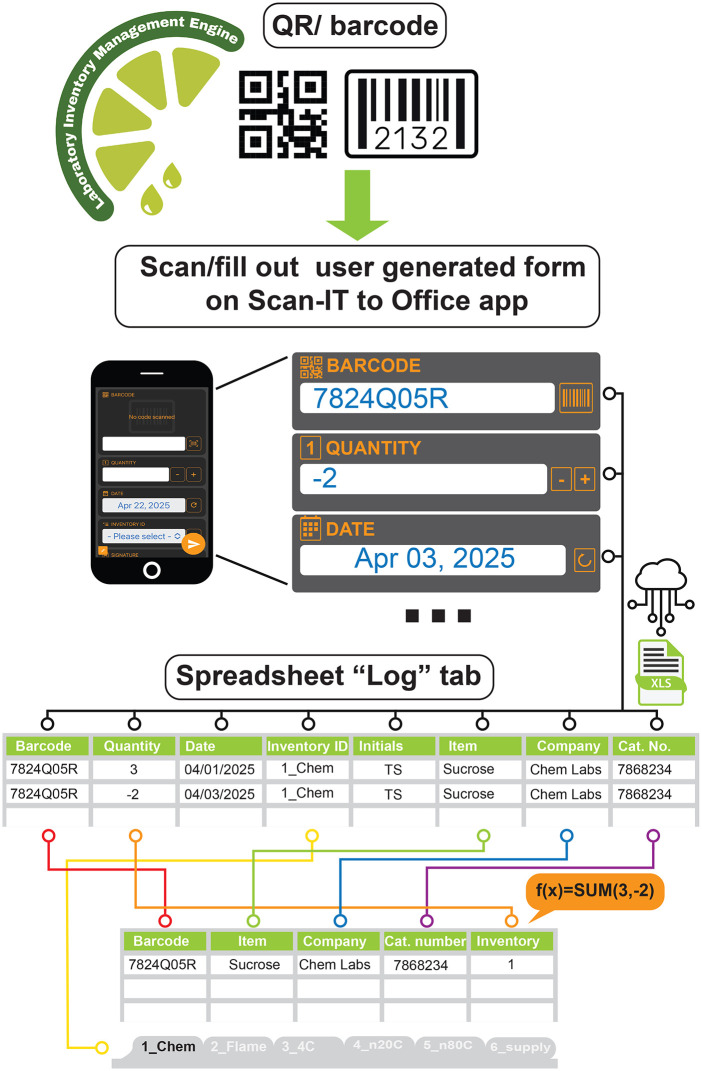
Workflow summary of LIME. The Scan-IT to Office^®^ phone/tablet application scans the item barcode. Once the barcode is read, the user fills the rest of the information fields. This information is transferred to the in-the-cloud spreadsheet log tab as a timestamp. The categorical spreadsheet tabs (e.g., “1_Chem”, “2_Flame”, etc.) tally the items based on their net quantity by calculating the net additions and subtractions of each item.

LIME works via communication between the mobile app as the input node and the in-the-cloud spreadsheet as the processing and data storage element (Fig 1). Through their mobile devices, users download an app, Scan-IT to Office^®^, to establish a connection with the spreadsheet located in a cloud storage service selected by the user. Through the app, the user creates a “form” with a series of varying inputs at their needs. Along with the inventory template, the users also receive a set of instructions guiding them on how to create the forms. Complete user documentation is available in the Supplemental Material S1 File.

In the Excel^®^/Google Sheets^®^ spreadsheet, a sheet titled “Log” keeps the “checks and balances” of the inventory. Among one of the input fields indicated on the forms, users create an “inventory ID” which serves to group/tag items that are being logged into user predefined categories such as the physical location of a set of items.

**Fig 2:**
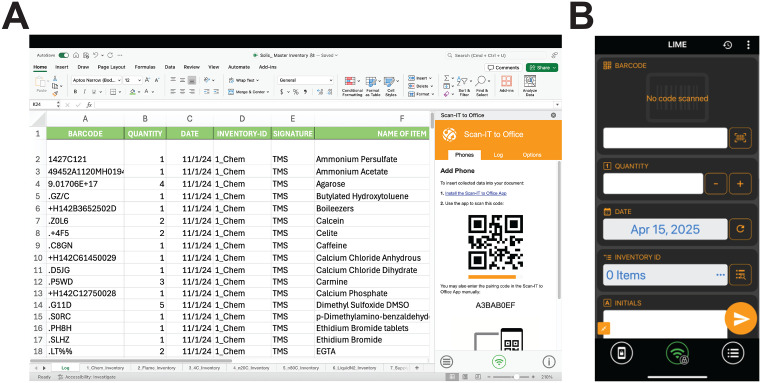
Visual appearance of LIME. (A) The LIME template on MS Excel® and (B) the form on the Scan-IT to Office^®^ app. Google Spreadsheet and Android® equivalents are not shown as they are visually similar.

The formulas implemented here on the spreadsheet further organize the materials into different catergorical tabs at the user’s discretion (e.g., chemical tab, −20 ºC tab, etc.) (Fig 2). Once the tabs have been decided, one must adjust the tab identifier name withing the formula from “1_Custom” to the desired element name in the spreadsheet tab that will match the name to be listed in the phone app. For example, if one tab is declared to be the chemicals tab, it could be written as “1_Chem” in the spreadsheet formula and the form entry named "INVENTORY ID" on the app. The usefulness lies in organizing the inventory into different sheets that may represent the physical location to these items. This facilitates the location of an item, as it sorts items based on their uniquely assigned barcode number. Barcode systems offer users an advantage in efficiency and fast acqusition by scanning devices, while simultaneously improving accuracy [4].

### Excel^®^ formulas


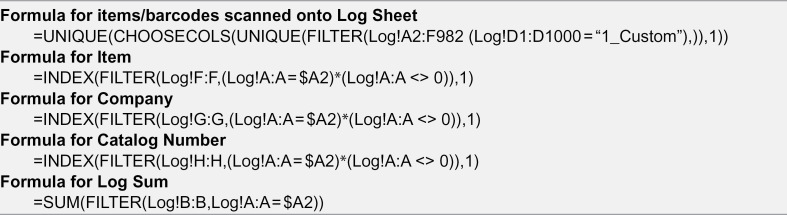


### Google Sheets^®^ Formulas


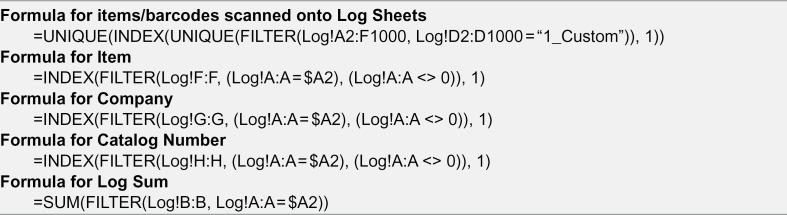


One benefit of the formula is that one only has to input the name, company, and catalog number the first time a particular item is scanned. In a scenario where one scans a barcode for a chemical that has already been logged in (e.g., if more has been ordered or if the item has run out), then one only needs to input the updated quantity added/removed, date, inventory ID, and initials as the other entries are not needed to perperly allocate the item in the assigned tab. This formula works by matching barcodes and automatically updates the new inventory number. The key feature to remember is that for the same item, the barcodes must match the original item to be properly categorized.

In the app, the history option gives access to every log that was inputted and the status of them (e.g., two green check marks means that the information was sent to the spreadsheet; two red check marks means the information failed to be sent). This is a unique aspect that provides greater security in the reduction of mistakes. Additionally, when an item is scanned, the app will save the input, even without a connection, and store it until the connection is restored, at which point it is sent..

## User survey

The survey was done using Qualtrics®. The survey study was approved by the Florida State University Institutional Review Board (IRB) and the study was registered under the identification number STUDY00005647. Participant recruitment for the survey was conducted from March 1 to April 16, 2025, during which eight participants were enrolled. Verbal consent was obtained and documented in the presence of a witness prior to system testing. All data were collected anonymously to ensure participant confidentiality and were accessed only after the completion of all surveys, which was after April 16^th^, 2025.

## Results and discussion

Here, LIME is presented as a free, open-source software tool designed to improve laboratory inventory management by reducing tracking time, improving accuracy, and minimizing reagent shortages. LIME offers substantial improvements over existing inventory management systems by prioritizing simplicity and combining advanced features like barcode integration and visible alerts with a user-friendly interface. Data entry is simplified through QR and barcode scanning, which reduces manual error and provides dynamic updates to inventory databases [6]. Custom fields in the form allow users to classify and separate inventory by lab-specific categories, improving searchability and organization.

An often-overlooked advantage of this approach is accessibility. Because LIME relies on universally available platforms and mobile technology, it garners a strong potential for wide adoption, not only in academic labs but also in startups and institutions with limited resources. Research shows that mobile-centric systems offer ergonomic, scalable, and cost-efficient solutions that align well with modern digital environments [7]. To demonstrate LIME’s effectiveness, a user guide was created and paired with a downloadable spreadsheet template (See Supplemental Material S1 File). This system was deployed across a number of research laboratories. User experience, perceived utility, and proficiency were measured through a series of follow-up questions. The survey revealed a net positive reception for LIME (Fig S1-S7 in S2 File), with 87.5% as moderately easy to set up (Fig S1 in S2 File), 75% describing it as extremely accurate and/or very accurate (Fig S4 in S2 File), and 75% describing that they will continue to use LIME (Fig S7 in S2 File). This suggests that the system has favorable functionality. Importantly, most users indicated that this system is on par or superior to their current laboratory inventory management system (Fig S3 in S2 File). This is noteworthy as the goal of this system is to offer a freely accessible inventory management system that can be adopted with minimal training.

The ability to track usage patterns and visualize data in real time provides researchers with the tools needed for better budgeting and resource allocation. Its open-source and multi-platform nature ensures that it can be adapted to suit various laboratory types and scales, with the potential for continuous improvement through community contributions. With its flexible, scalable design and open-source license, LIME represents a valuable contribution to laboratory efficiency and resource management. Future developments will focus on expanding its features based on user feedback and ensuring its long-term viability through community-supported updates.

Despite all the benefits highlighted, LIME has a number of limitations to consider. First, the connectivity to the phone app cloud is slow due to the latent time of the cloud server refresh. Second, the spreadsheets are prone to having incorrect information being inserted by inexperienced users. It is highly recommended that the management of supply inputs and outputs from LIME be done by full-time laboratory members rather than part-time students to maintain concistency and an up-to-date inventory. Third, since the version that is used for the app is free, at random times, a prompt message stating “DEMO - Please subscribe to use full version” appears into a cell in the spreadsheeet, which does not input the data that was recently entered. If this happens, one must rescan the item again. Other errors such as “Internal error” appear, but the data usually is transfered to the spreadsheet. If not, the ‘History’ option on the app will save that input and will give the user the option to resend. In other cases, one can use the ‘log’ tab on the spreadsheet as a version control to check for incorrect information inputs. Overall, these are limitations that are part of the compromise of making a system free and accessible to use from widely available software tools.

## Supporting information

S1 File contains user instruction manual to implement LIME using Excel^®^ or Google Sheets^®^ as well as the iPhone^®^ and Android^®^ for the Scan-IT to Office^®^ phone app. (DOCX)

S2 File contains survey result figures S1-S7. (DOCX)
