# Supplementary material for: Laboratory Inventory Management Engine (LIME): A free tool for managing laboratory inventories via barcode scanning and automatic cloud-based spreadsheet integration
Source: PLoS One. 2026 May 22;21(5):e0336412. doi: 10.1371/journal.pone.0336412 (PMC13196951; doi:10.1371/journal.pone.0336412)

**Supplemental Material S1: Instructions to install Phone app and spreadsheet setup for Excel and Googlesheets**

**(Salinas et al. 2025)**

1. **Adding the Scan-IT to Office Add-In to Excel.**
2. Open the downloaded Excel file template which was shared with you.
3. Go to the file, click on “Get Add-ins.”
4. Under Get Add-ins, click on “More Add-ins” and use the search option to look for “Mobile Data Collection - Scan-IT to Office.”
5. **Connecting A Device onto the Spreadsheet**
6. Once you have added Scan-IT to Office onto the spreadsheet, it will be listed under “My Add-ins.” Click on it. Upon clicking, a side window should open, allowing the connection for devices and log history.
7. Click on “Add Phone”
8. Download the app “Scan-IT” onto your cellular device.
9. Open the app and tap on the bottom middle icon. It should look like this:

                                                                      
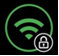


1. Tap on “+ Add Connection”
2. Tap on the “Microsoft Word/Excel option”. This should prompt a screen allowing you to scan the QR code on the spreadsheet.

**Once you have scanned the QR code, you have successfully connected your device.**

1. Click “Options” and scroll until you see the following


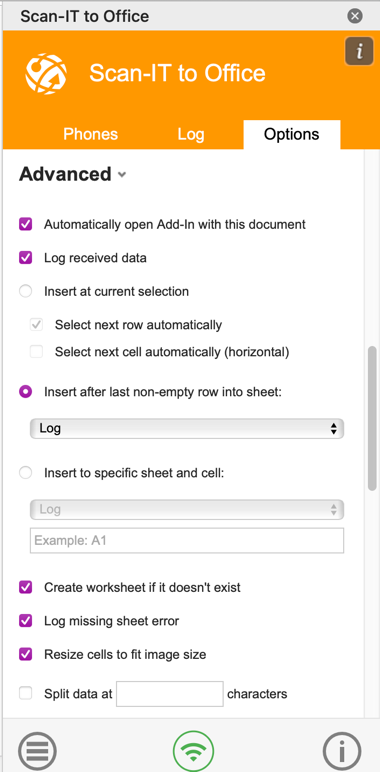


1. Make sure to click the option that says “Automatically open Add in with this document” and “Insert after last non-empty row into sheet”.

***Note:*** *If you do* ***not*** *press* ***“Automatically open Add-in with this document,”*** *you will need to manually open the add-in each time to ensure your phone connects to the sheet. A* ***green WiFi symbol*** *on both your phone and the spreadsheet will indicate a successful connection.*

1. To generate a log, you must create a form on the app. The instructions will be as follows:
2. **Creating A Form on Scan-IT to Office**
3. Open the “Scan-IT to Office” app. Tap on the far-right icon. It should look like this:


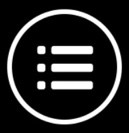


1. You should be prompted to a screen that shows numerous forms.


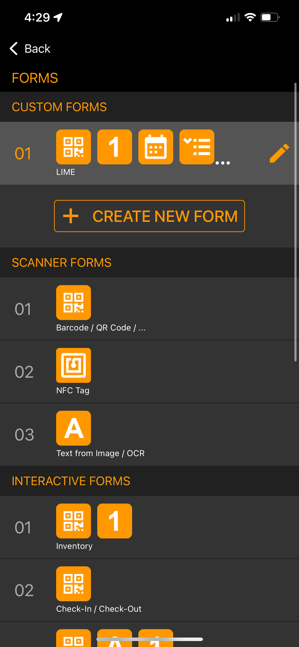


1. Under “CUSTOM FORMS”, tap on “+ CREATE NEW FORM”
2. Title the Form
3. The Form will consist of 8 fields.

***NOTE:*** *The* ***order is crucial*** *— data will transfer* ***exactly in this sequence****, which must match the order on the* ***“LOG”*** *sheet on the Excel template*. *These are also our examples of the kind of data we want to have. Feel free to customize based your needs*

1. The first field is a “Barcode” field. Under “Name” write “BARCODE”

- This one should appear automatically

1. Click on “+ ADD FIELD”
2. The second field is a “Number” field.


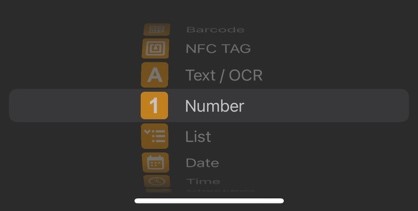


- Under “Name” write “QUANTITY”

1. Click on “+ ADD FIELD”
2. The third field is a “Date” field.


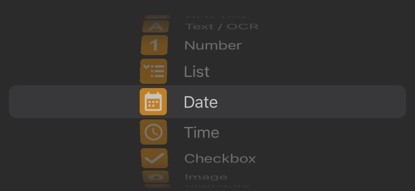


- Under “Name” write “DATE”

1. Click on “+ ADD FIELD”

The fourth field is a “List” field.


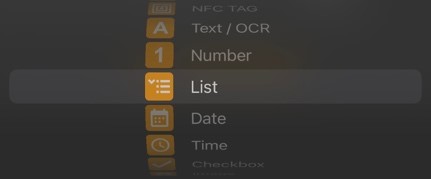


Under “Name” write “INVENTORY ID”

1. Under Elements:


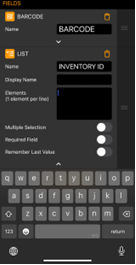


 Type

1. 1_Chem

Click “Return” on your keyboard

1. 2_Flame

Click “Return” on your keyboard

1. 3_4C

Click “Return” on your keyboard

1. 4_n20C

Click “Return” on your keyboard

1. 5_n80c

Click “Return” on your keyboard

1. 6_Supply

***Note:*** *These element names are* ***meant to be customized*** *based on your needs and are the tab names on the spreadsheet. Please ensure that the* ***Inventory ID*** *names (e.g., 1_Chem, 2_Flame, etc.) on the* ***app*** *match the corresponding* ***sheet Tabs*** *and* ***formulas*** *in Excel/GoogleSheets.*

1. The fifth field is a “Text / OCR” field.


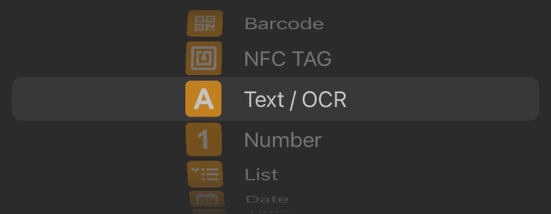


- Under “Name” write “INITIALS”

1. The sixth field is a “Text / OCR” field.


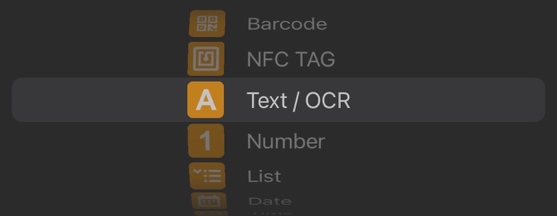


- Under “Name” write “NAME OF ITEM”

1. The seventh field is a “Text / OCR” field.


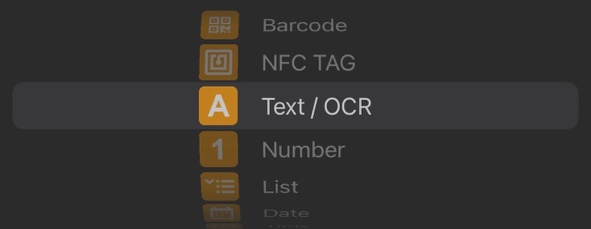


- Under “Name” write “COMPANY”

1. The eighth field is a “Text / OCR” field.


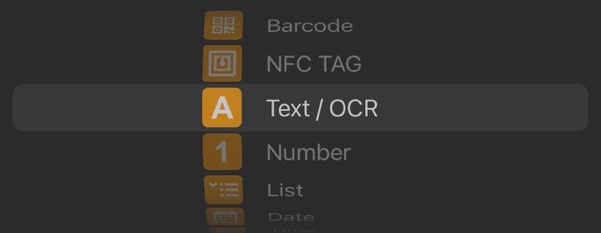


- Under “Name” write “CAT NO.”

1. Once completed, click “Back”. Don’t worry, the form will save.

***NOTE:***

- *You will only have to input the name, company, and catalog number the first time you ever scan an item.*
- *In a scenario where you scan a barcode for a chemical that has already been logged in, then you only need to input the new quantity, date, inventory ID, and signature. The formula works so that it will be able to match barcodes if they are the same and automatically update the new inventory number.*

1. **Generating Inventory Inputs**
2. Start on the Sheet titled “Log”
3. Click on the uppermost left cell
4. To find your form on the app, click the far-right icon.
5. Click on your form. It should appear as this:


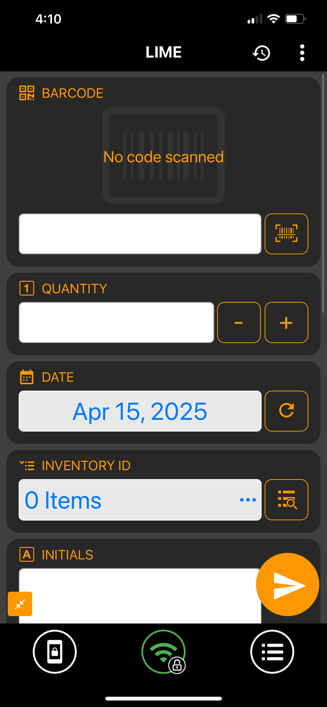


1. Use your connected device to fill out the form made in the prior step.
2. Once the fields have been filled, use the arrow to send the log to the spreadsheet.
3. The middle icon will either turn green, meaning that your data is already on the spreadsheet, or orange, meaning that it is pending. Once you open your Excel/Google Sheets spreadsheet, the data will go through in approximately 1 minute. Please resist the temptation to refresh the sheet. As this will erase the data.
4. **Backlogging:**


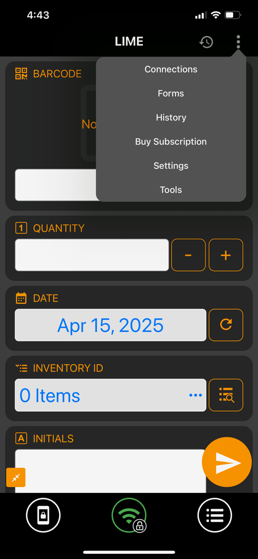


 A key feature of this app is that it provides a ‘History’ option where it shows all the data that has been sent. Green arrows mean that the data successfully went through. Gray arrows vary in what they mean. Sometimes they go through and in case it doesn’t, if you click on the data entry, it will let you resend the data. Red arrows mean there was an error, and you must rescan.

*Note: An “internal error” may occasionally appear — this is a known issue with the app.* 
*In most cases, the data still goes through successfully, but it will appear as grey checkmarks on the app.*


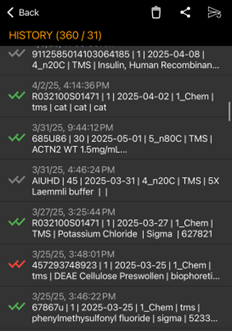


ADDITIONAL NOTES/SUGGESTIONS

- Forms are extremely malleable. Allowing for the ability to create custom forms with any combination of input fields.
- If you come across an item with no barcode, you can generate a barcode for that item with websites online, then print it out and tape it.
- We use a label printer from the brand BROTHER. The label printer is connected to an app which can generate and print out barcodes, QR codes, and other similar things.
- For duplicate chemicals, you can scan one barcode and then duplicate that barcode for the rest of the chemicals.
- To do this:
- Scan the designated barcode into the app and copy the translated numbers/characters it generated.
- Paste that information into the barcode generator to generate the same barcode. A similar approach can be followed for QR codes.

**Uploading the LIME template onto Google Spreadsheets**

1. Open the downloaded Excel LIME template which was shared with you.
2. Go to Google Drive, press “+New” and upload the Excel LIME template. Double-click on it and it will open into Google Sheets.
3. Go to files and save as Google Sheets. The Google Sheets version will open in another tab.
4. Go to files and save. Copy and paste the following formulas on the Tab sheets. For the first barcode tab on your sheet, you only have to put in the formula once. DO NOT DRAG DOWN. For the other items, you must drag down.

- **Formula for items/barcodes scanned onto Log Sheet**
- =UNIQUE(INDEX(UNIQUE(FILTER(Log!A2:F1000, Log!D2:D1000 = "1_Chem")), , 1))

- **Formula for Custom Header**

=INDEX(FILTER(Log!F:F, (Log!A:A = $A2) , (Log!A:A <> 0)), 1)

- **Formula for Custom Header**

=INDEX(FILTER(Log!G:G, (Log!A:A = $A2) , (Log!A:A <> 0)), 1)

- **Formula for Custom Header**

=INDEX(FILTER(Log!H:H, (Log!A:A = $A2) , (Log!A:A <> 0)), 1)

- **Formula for Log Sum**

=SUM(FILTER(Log!B:B, Log!A:A = $A2))

1. **Adding the Scan-IT to Office Add-On to Google Sheets**
2. Go to the Extensions and click on “Add-ons.”

- Under Get Add-ons, click on “Get Add-ons” and use the search option to look for “Mobile Data Collection - Scan-IT to Office.” You must always open this add-on to connect your phone to the spreadsheet. It will appear under ‘Extensions’ all the way at the bottom.


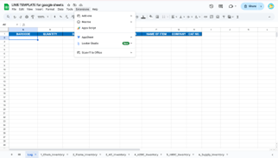


1. **Adding the Scan-IT to Office Add-In to Excel.**
2. Open the downloaded Excel file template which was shared with you.
3. Go to the file, click on “Get Add-ins.”

- Under Get Add-ins, click on “More Add-ins” and use the search option to look for “Mobile Data Collection - Scan-IT to Office.”

1. **Connecting A Device onto the Spreadsheet**
2. Upon clicking, a side window should open, allowing the connection for devices and log history
3. Click on “Add Phone”

- Download the app “Scan-IT” onto your device.
- Open the app and tap on the middle icon. It should look like this:

  
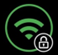


III. Tap on “+ Add Connection”

1. Tap on the “GoogleSpreadsheet”. This should prompt a screen allowing you to scan the QR code on the spreadsheet.

**Once you have scanned the QR code, you have successfully connected your device.**

1. Click “Options” and scroll until you see the following


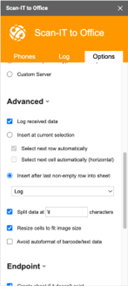


1. Make sure to click the option that says “Insert after last non-empty row into sheet”

To generate a log, you must create a form on the app. The instructions will be as follows:

1. **Creating A Form on Scan-IT to Office**
2. Open the “Scan-IT to Office” app. Tap on the far-right icon. It should look like this:

    
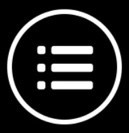


1. You should be prompted to a screen that shows numerous forms.
2.
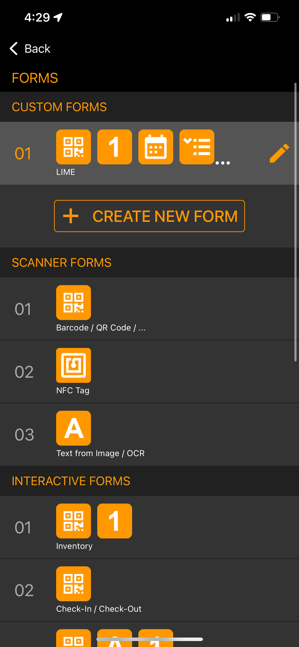

3. Under “CUSTOM FORMS”, tap on “+ CREATE NEW FORM”
4. Title the Form
5. The Form will consist of 8 fields.

***NOTE:*** *The* ***order is crucial*** *— data will transfer* ***exactly in this sequence****, which must match the order on the* ***“LOG”*** *sheet on the Google Sheets template*.

1. The first field is a “Barcode” field. Under “Name” write “BARCODE”

- This one should appear automatically

1. Click on “+ ADD FIELD”
2. The second field is a “Number” field.


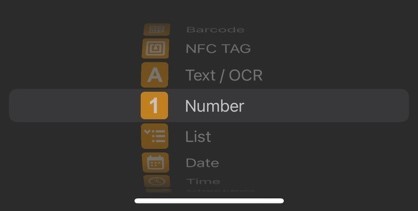


- Under “Name” write “QUANTITY”

1. Click on “+ ADD FIELD”
2. The third field is a “Date” field.


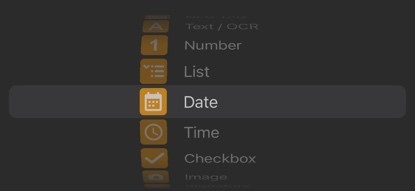


- Under “Name” write “DATE”

1. Click on “+ ADD FIELD”

The fourth field is a “List” field.


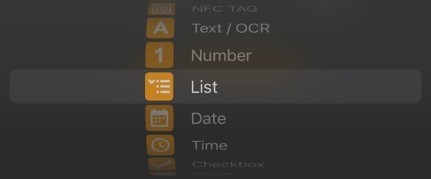


Under “Name” write “INVENTORY ID”

- Under Elements:

  
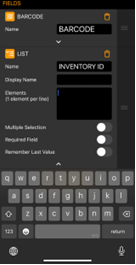


Type

1. 1_Chem

Click “Return” on your keyboard

1. 2_Flame

Click “Return” on your keyboard

1. 3_4C

Click “Return” on your keyboard

1. 4_n20C

Click “Return” on your keyboard

1. 5_n80c

Click “Return” on your keyboard

1. 6_Supply

***Note:*** *These element names are* ***meant to be customized*** *based on your needs. Please ensure that the* ***Inventory ID*** *names (e.g., 1_Chem, 2_Flame, etc.) on the* ***app*** *match the corresponding* ***sheet Tabs*** *and* ***formulas*** *in Google Sheets.*

1. The fifth field is a “Text / OCR” field.


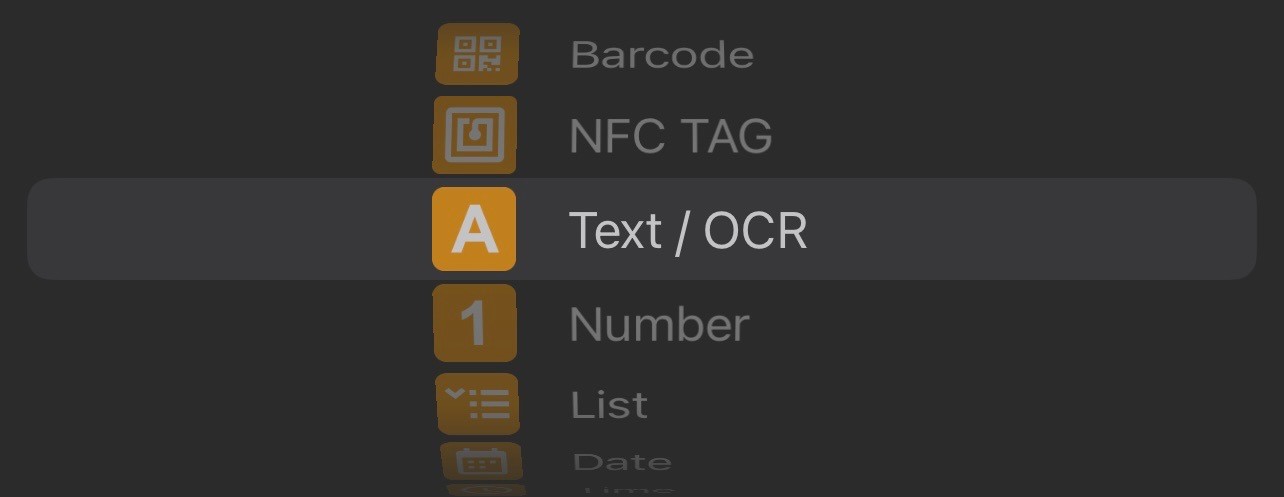


- Under “Name” write “INITIALS”

1. The sixth field is a “Text / OCR” field.


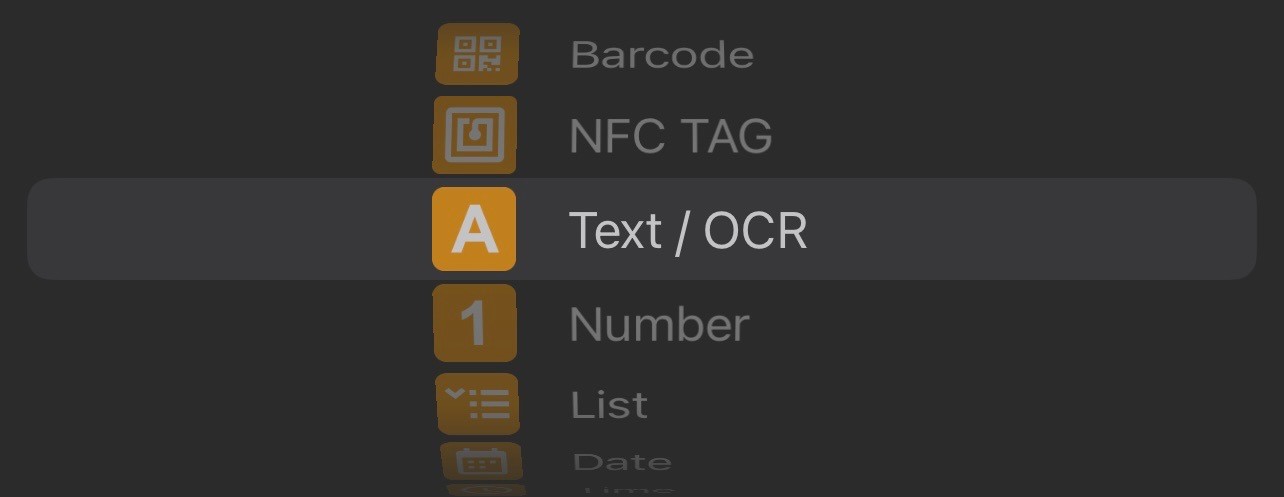


- Under “Name” write “NAME OF ITEM”

1. The seventh field is a “Text / OCR” field.


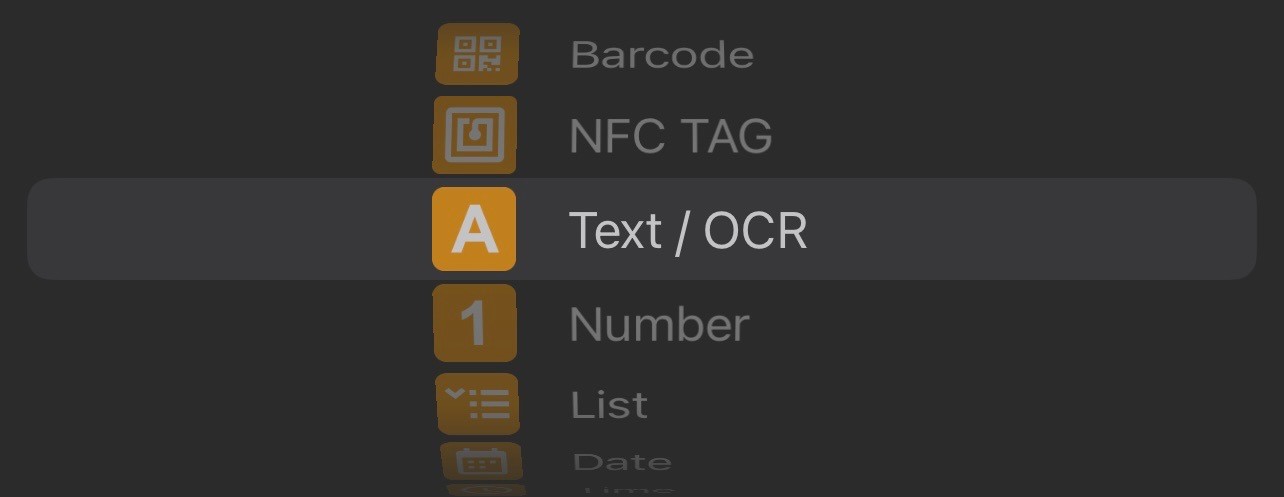


- Under “Name” write “COMPANY”

1. The eighth field is a “Text / OCR” field.


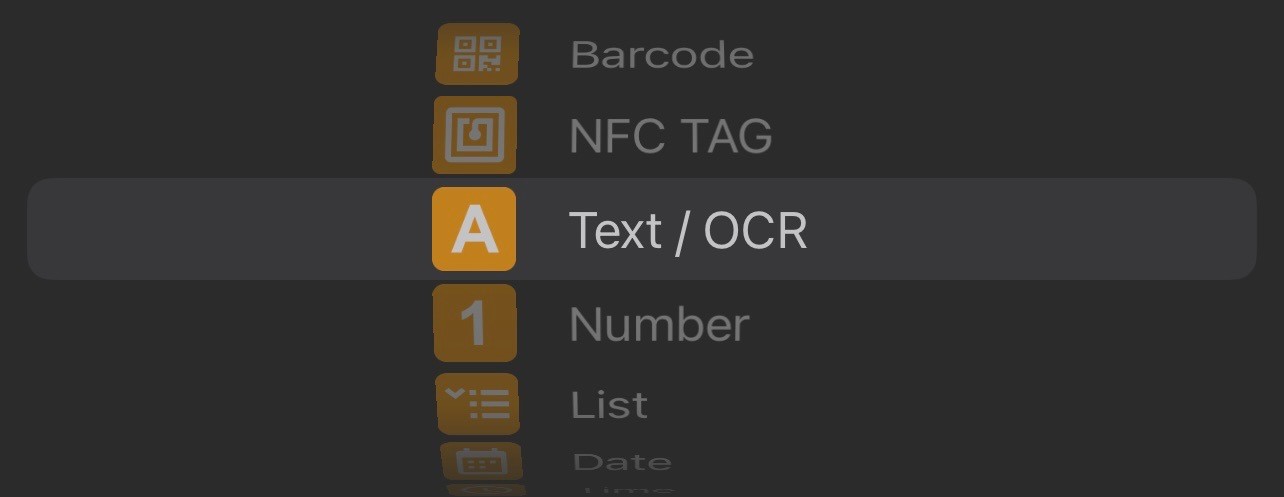


- Under “Name” write “CAT NO.”

1. Once completed, click “Back”. Don’t worry, the form will save 😊

***NOTE:***

- *You will only have to input the name, company, and catalog number the first time you ever scan an item.*
- *In a scenario where you scan a barcode for a chemical that has already been logged in, then you only need to input the new quantity, date, inventory ID, and signature. Our formula works so that it will be able to match barcodes if they are the same and automatically update the new inventory number.*

1. **Generating Inventory Inputs**
2. Start on the Sheet titled “Log”
3. Click on the uppermost left cell
4. To find your form on the app, click the far-right icon.
5. Click on your form. It should appear as this:


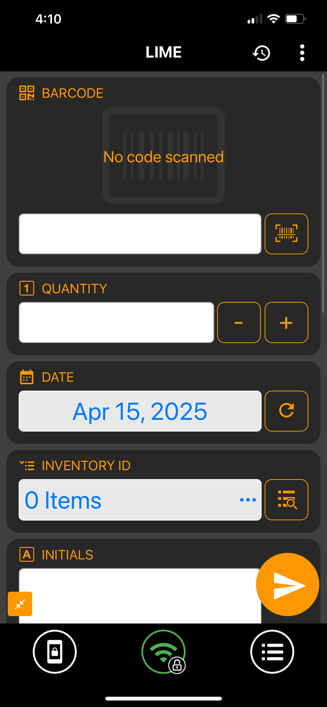
 

1. Use your connected device to fill out the form made in the prior step.
2. Once the fields have been filled, use the arrow to send the log to the spreadsheet.
3. The middle icon will either turn green, meaning that your data is already on the spreadsheet or orange, meaning that it is pending. Once you open your sheet, the data will go through in approximately 1 minute. Please resist the temptation to refresh the sheet. As this will erase the data.
4. **Backlogging:**


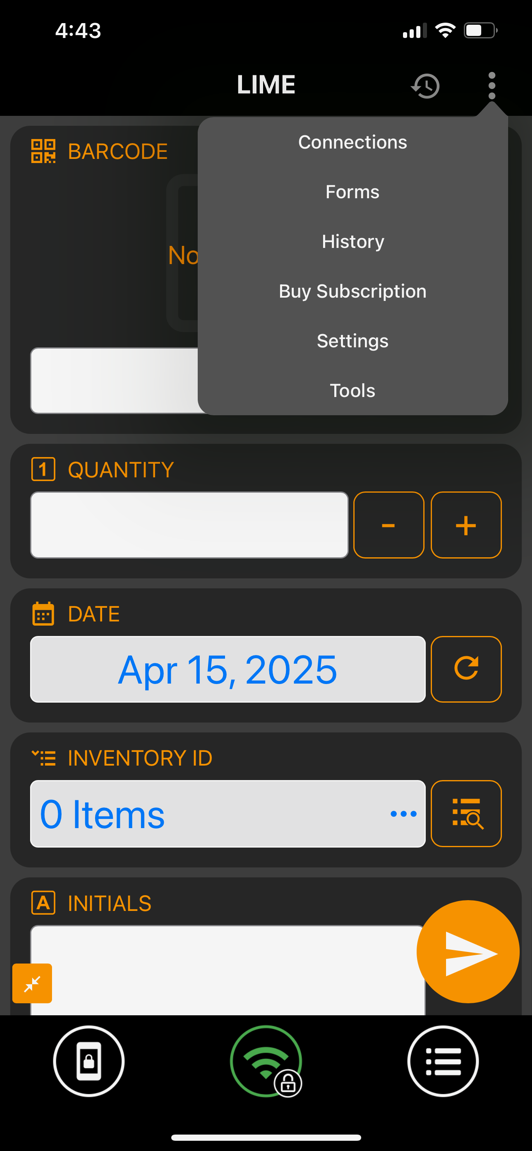


 A key feature of this app is that it provides a ‘History’ option where it shows all the data that has been sent. Green arrows mean that the data successfully went through. Gray arrows vary in what they mean. Sometimes they go through and in case it doesn’t, if you click on the data entry, it will let you resend the data. Red arrows mean there was an error, and you must rescan.

*Note: An “internal error” appears often and is just a fault of the app. Most of the time, the data goes through but on the app, it will appear as grey checkmarks.*


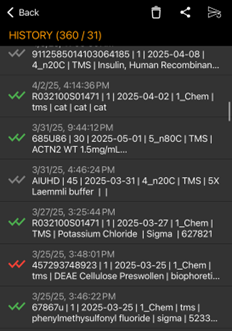

Supplement: S1 File — (DOCX) [file pone.0336412.s001.docx]
