# Supplementary material for: Laboratory Inventory Management Engine (LIME): A free tool for managing laboratory inventories via barcode scanning and automatic cloud-based spreadsheet integration
Source: PLoS One. 2026 May 22;21(5):e0336412. doi: 10.1371/journal.pone.0336412 (PMC13196951; doi:10.1371/journal.pone.0336412)
Supplement: S2 File — (DOCX) [file pone.0336412.s002.docx]

**Supplemental Material S2: Survey Results**

**(Salinas et al. 2025)**


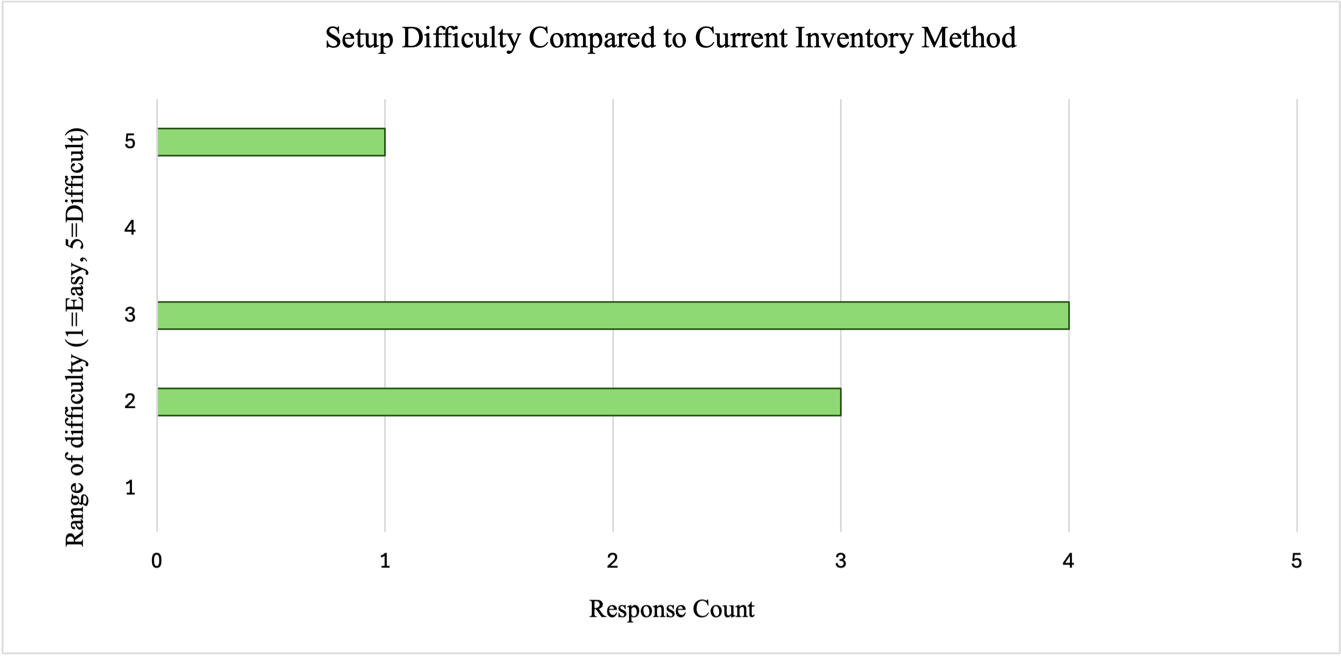


**Fig S1. Participant response addressing the setup difficulty.** The survey participants were asked how the setup difficulty of LIME was in comparison to their current inventory method. Responses were rated on a scale from 1 being *easy*, to 5 being *difficult* (N =8).


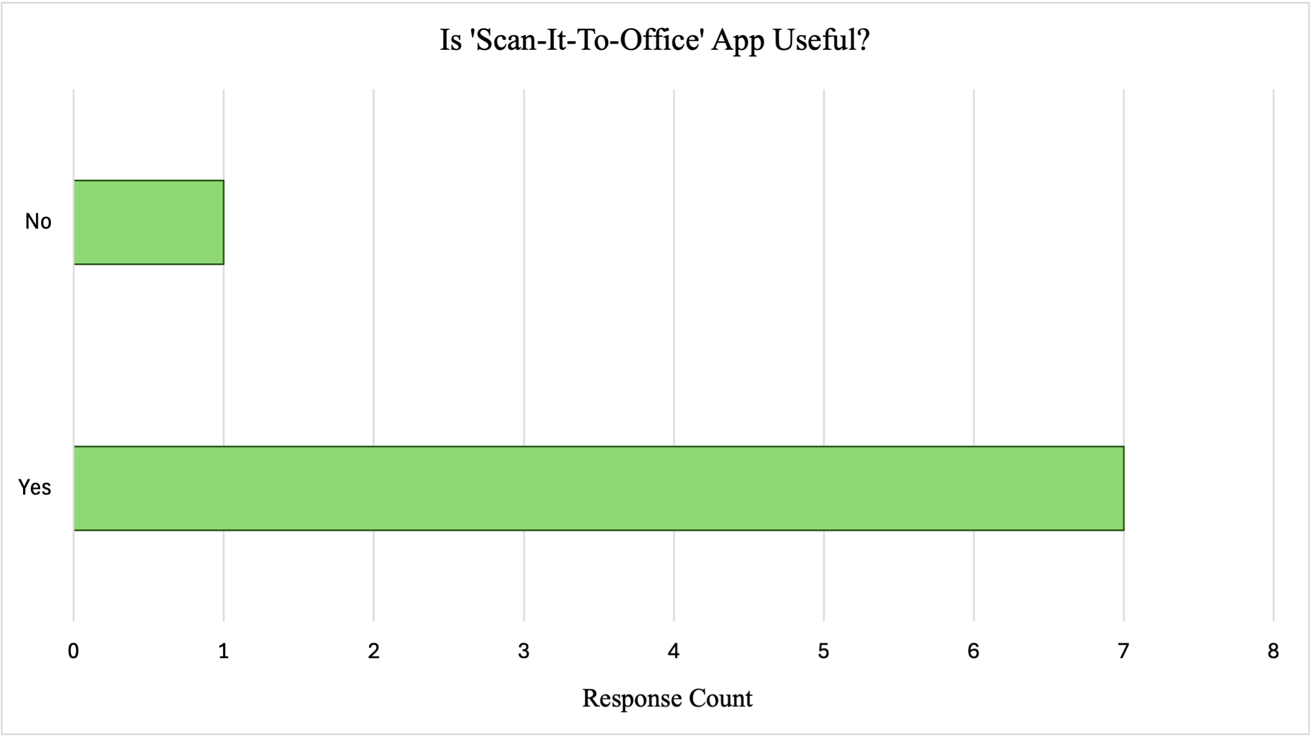


**Fig S2. Participant response regarding the perceived usefulness of the Scan-IT to Office addition.** Response options included *yes* and *no* (N =8).


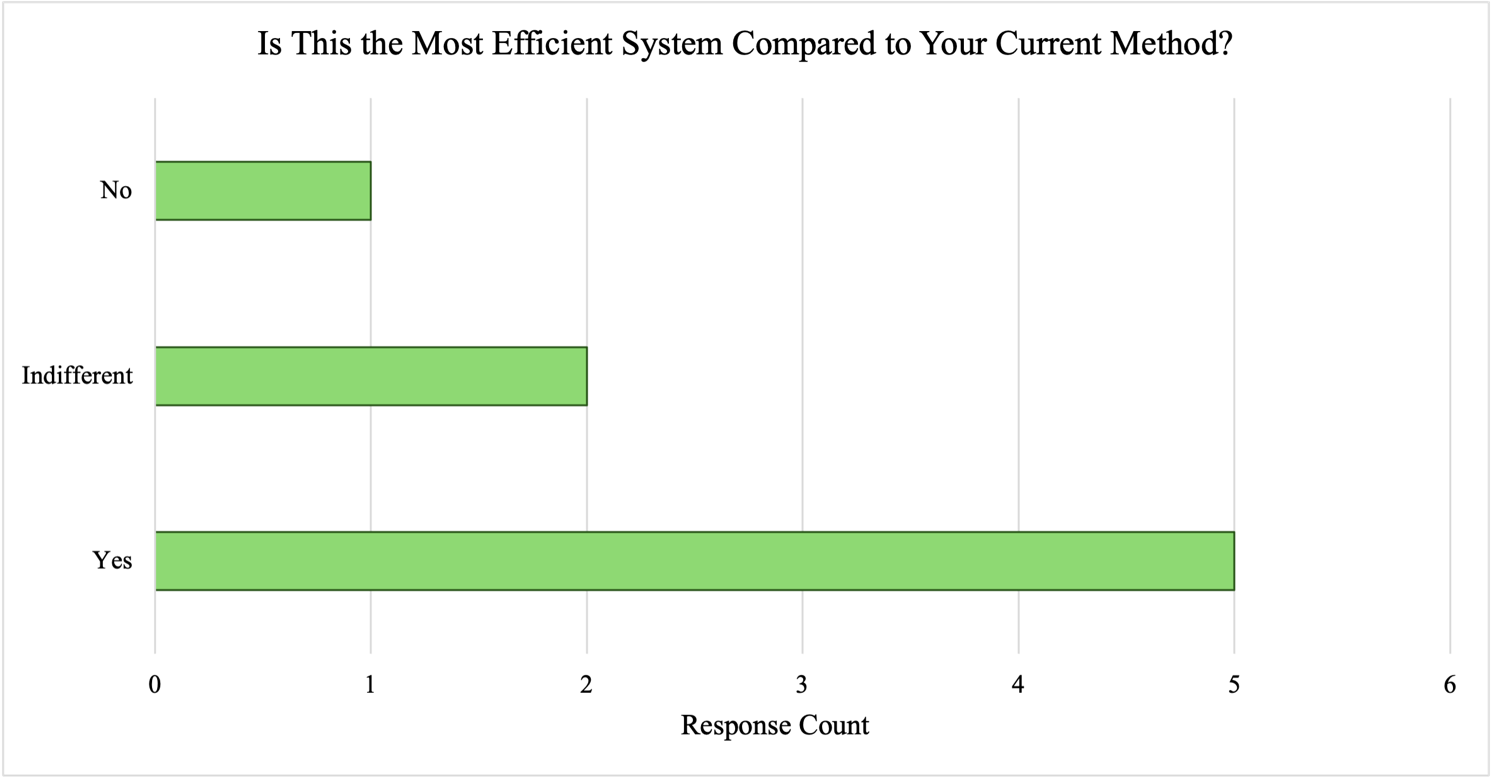
**Fig S3.** **Participant responses evaluating efficiency in relation to their current inventory system and the LIME system**. Participants were asked to compare their current lab inventory management system vs. LIME on their perceived operational efficiency (N = 8).


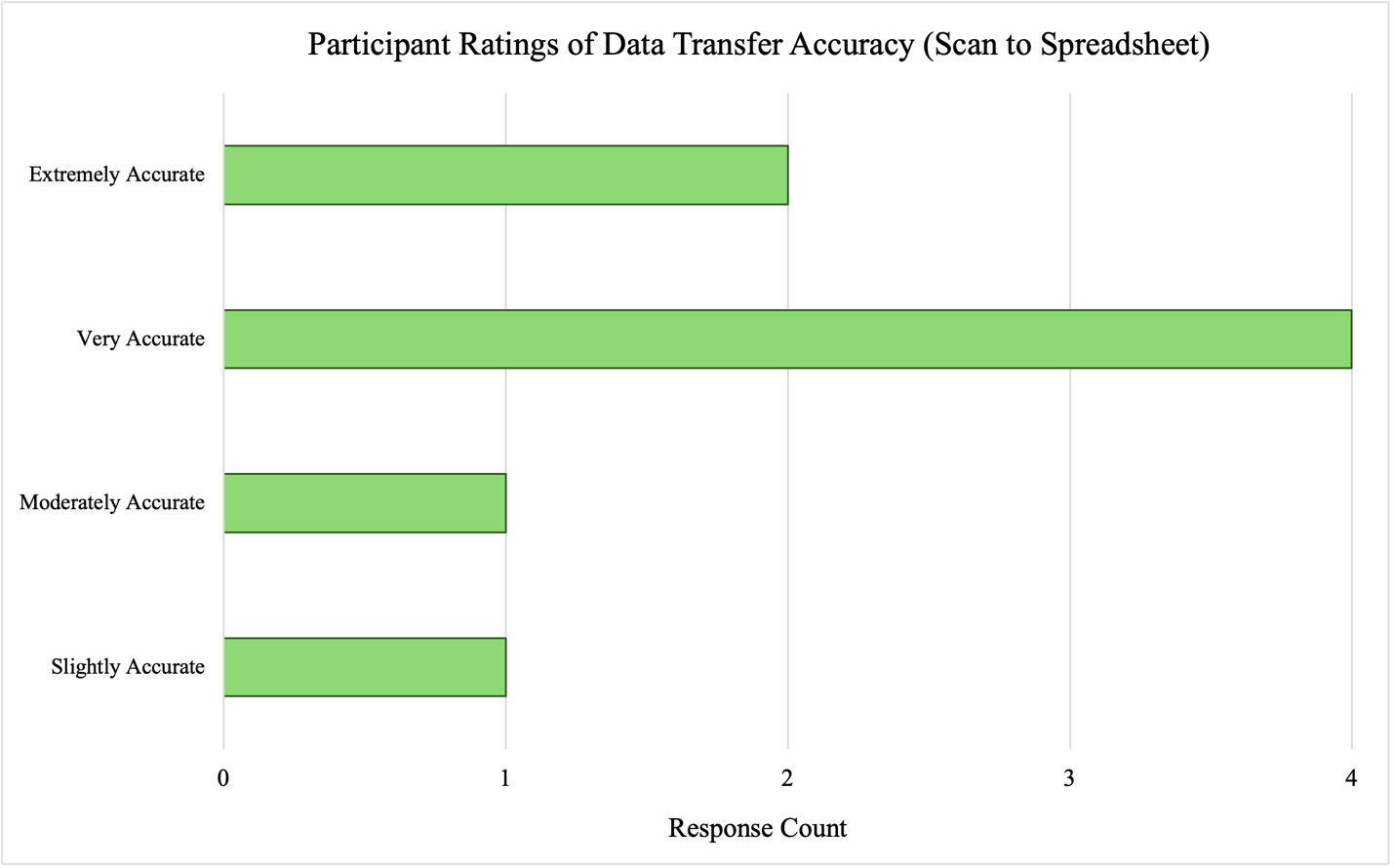
**Fig S4.** **Participant response addressing the accuracy of data transfer from the Scan-IT to Office app to the spreadsheet.** Responses were rated on a scale from slightly accurate to extremely accurate (N =8).


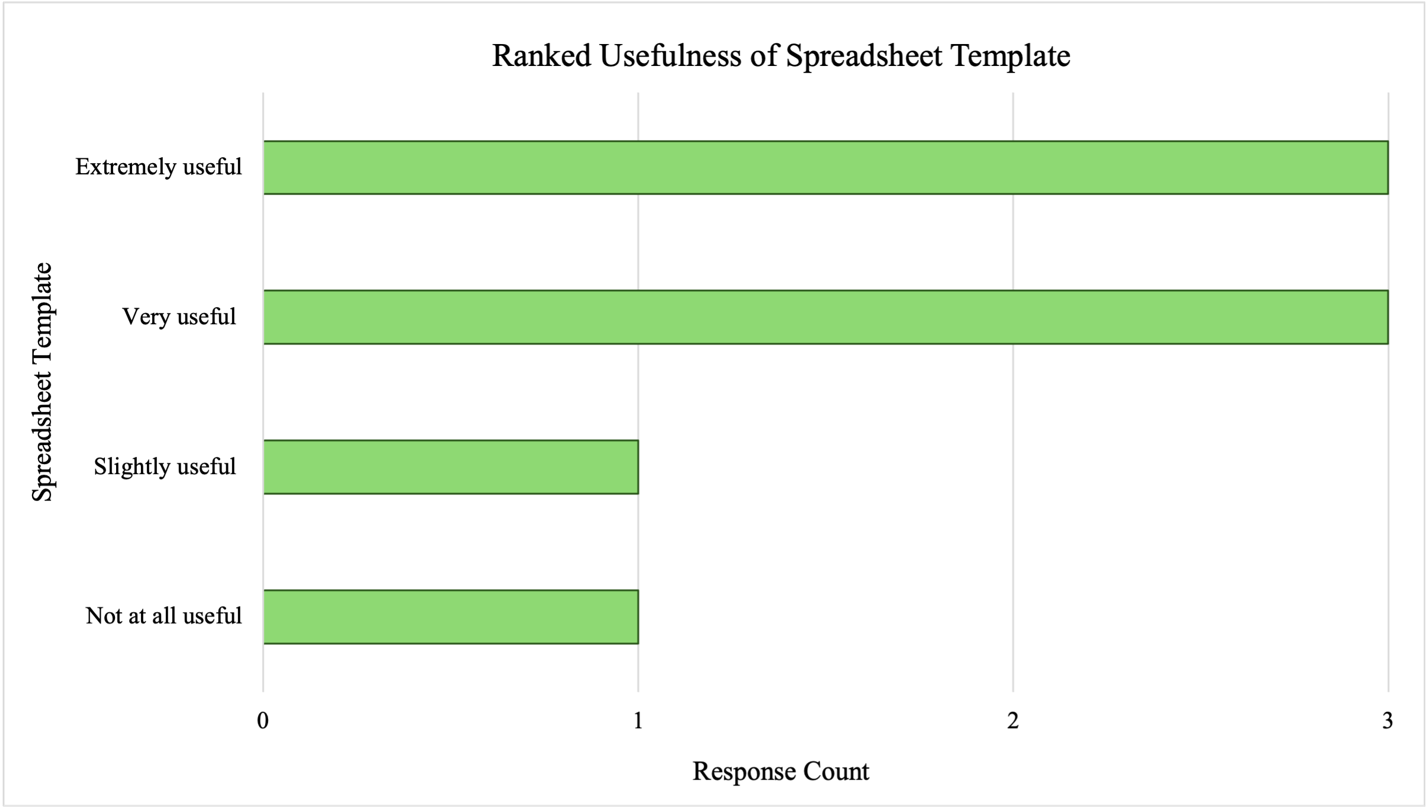
**Fig S5.** **Participant response regarding the perceived usefulness of the spreadsheet template.** Responses were rated on a scale from not at all useful to extremely useful (N =8).


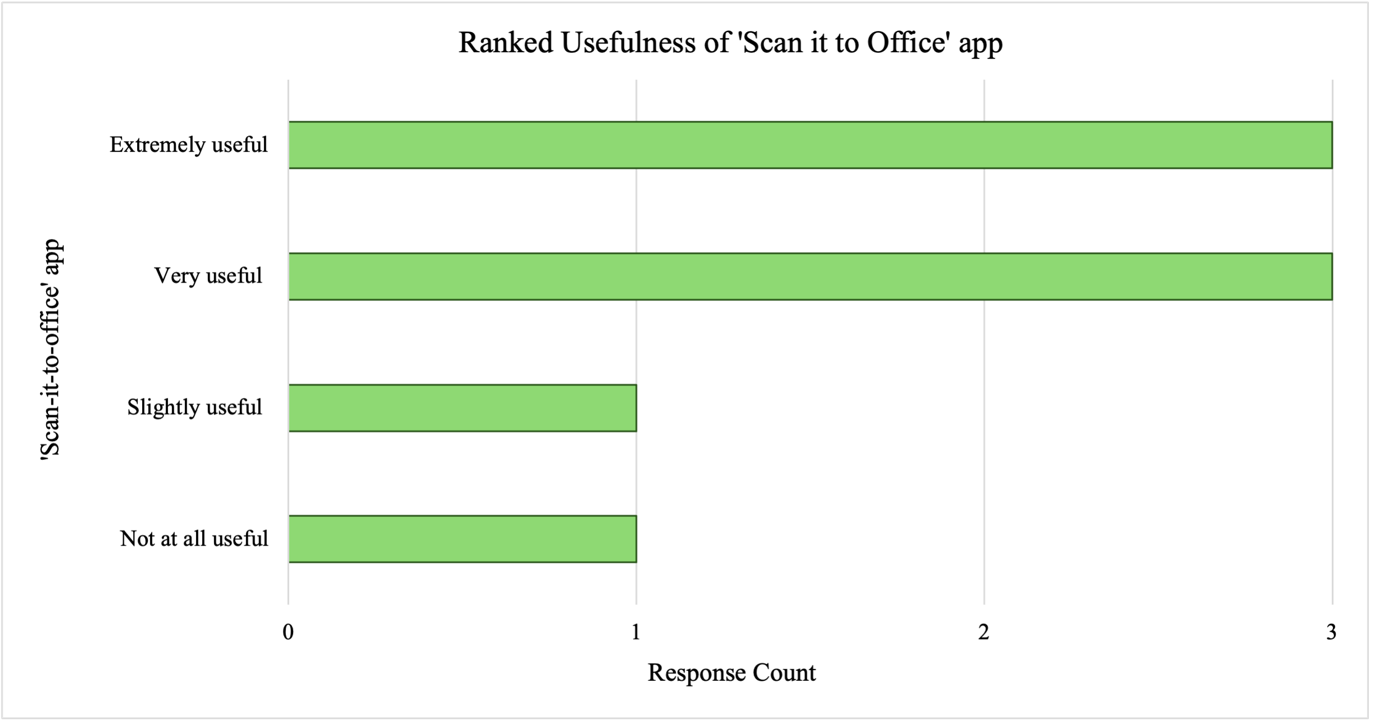


**Fig S6.** **Participant response regarding the perceived usefulness of the Scan-IT to Office app.** Responses were rated on a scale from not at all useful to extremely useful (N =8).


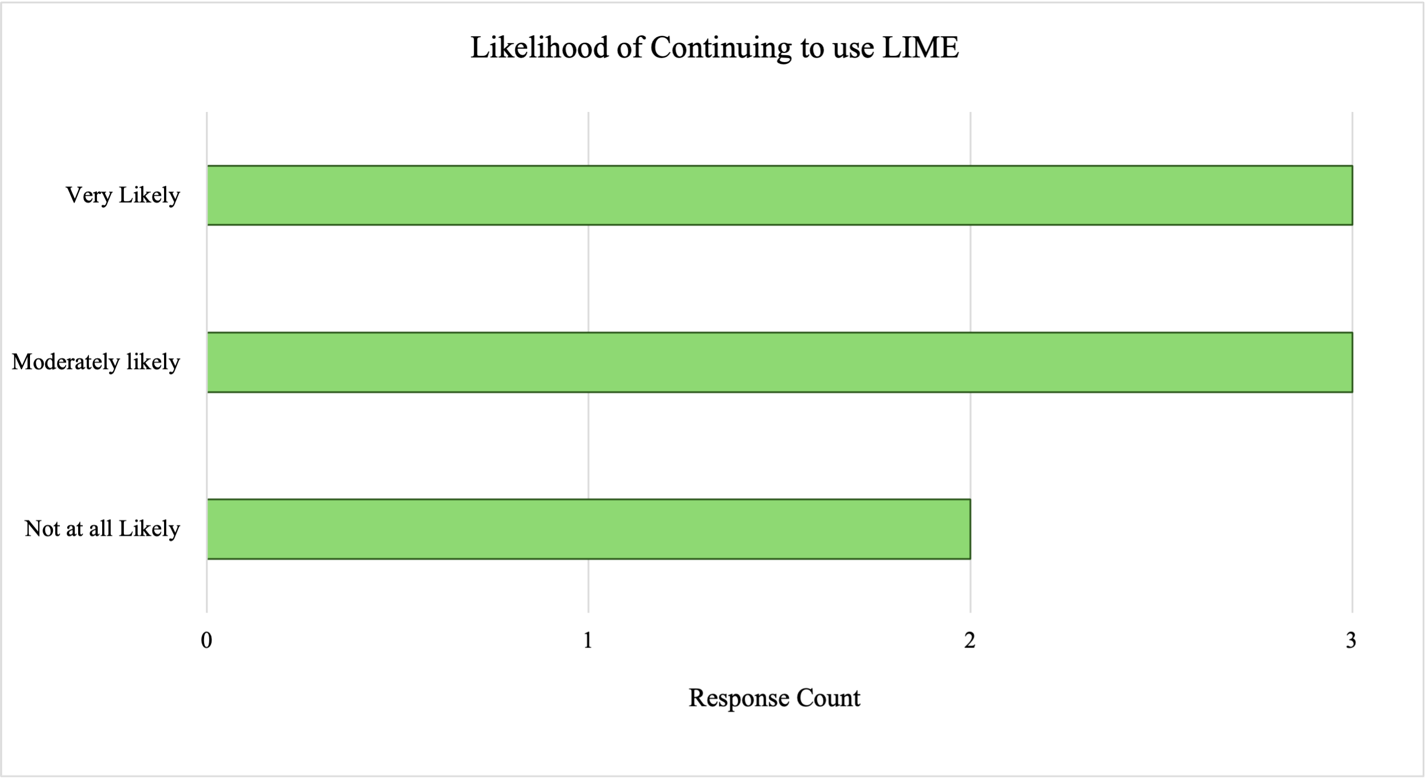
**Fig S7.** **Participant response regarding the likelihood of continuing to use LIME**. Responses were rated on a scale not at all likely to very likely (N=8).
